# Supplementary figures and images for: The mAB 13A4 monoclonal antibody to the mouse PROM1 protein recognizes a structural epitope
Source: PLoS One. 2022 Oct 10;17(10):e0274958. doi: 10.1371/journal.pone.0274958 (PMC9550058; doi:10.1371/journal.pone.0274958)

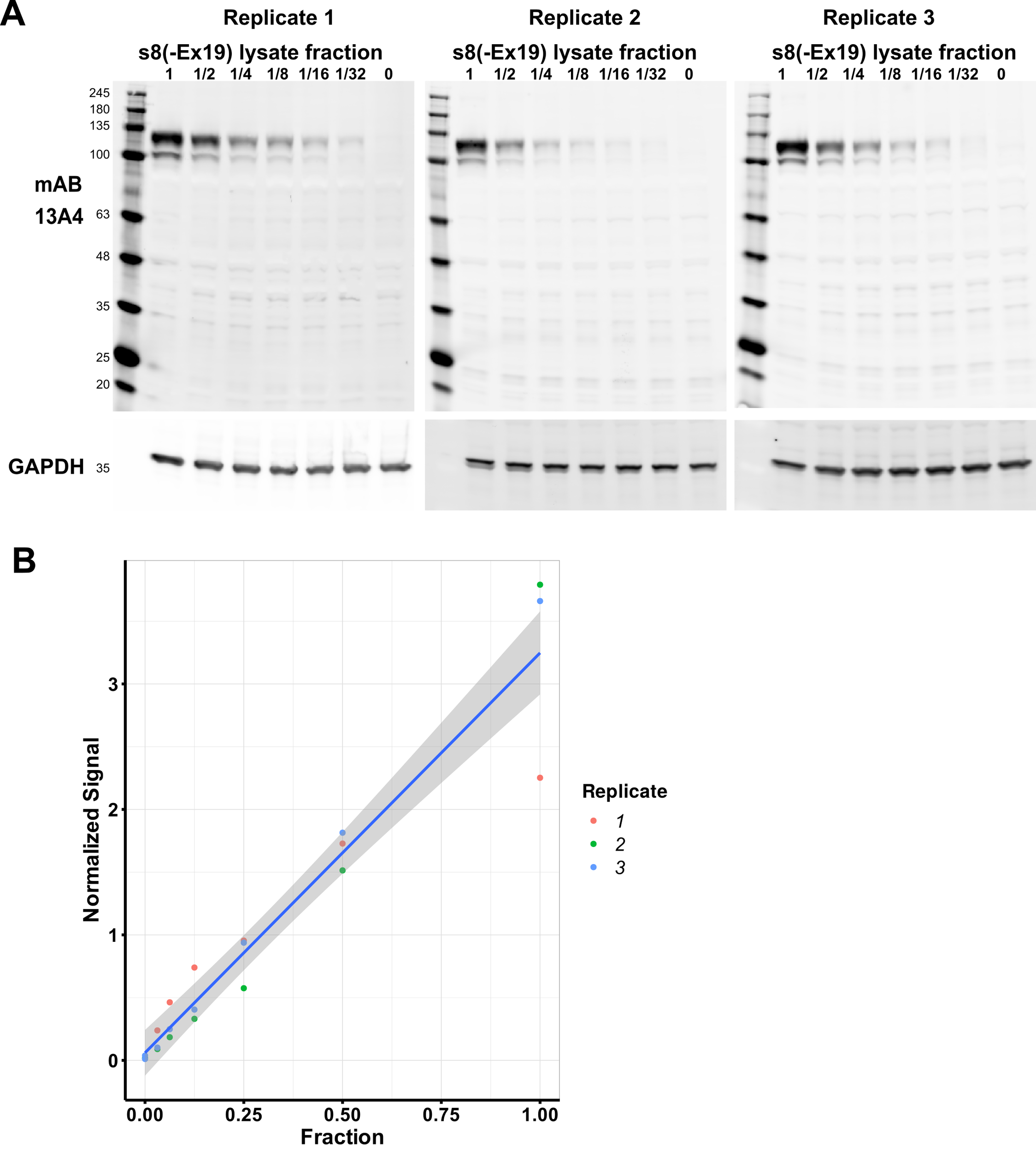

Supplement: S1 Fig — A) Western blot replicates. Serial dilution of extract expressing clone s8(-Ex19) with extract from cells transfected with an empty vector was probed by mAB 13A4. B) Plot showing scaled normalized signal intensities for each replicate and linear regression with 95% confidence interval. R2 = 0.93, p-value = 1.8*10−12. (TIF) [file pone.0274958.s002.tif]

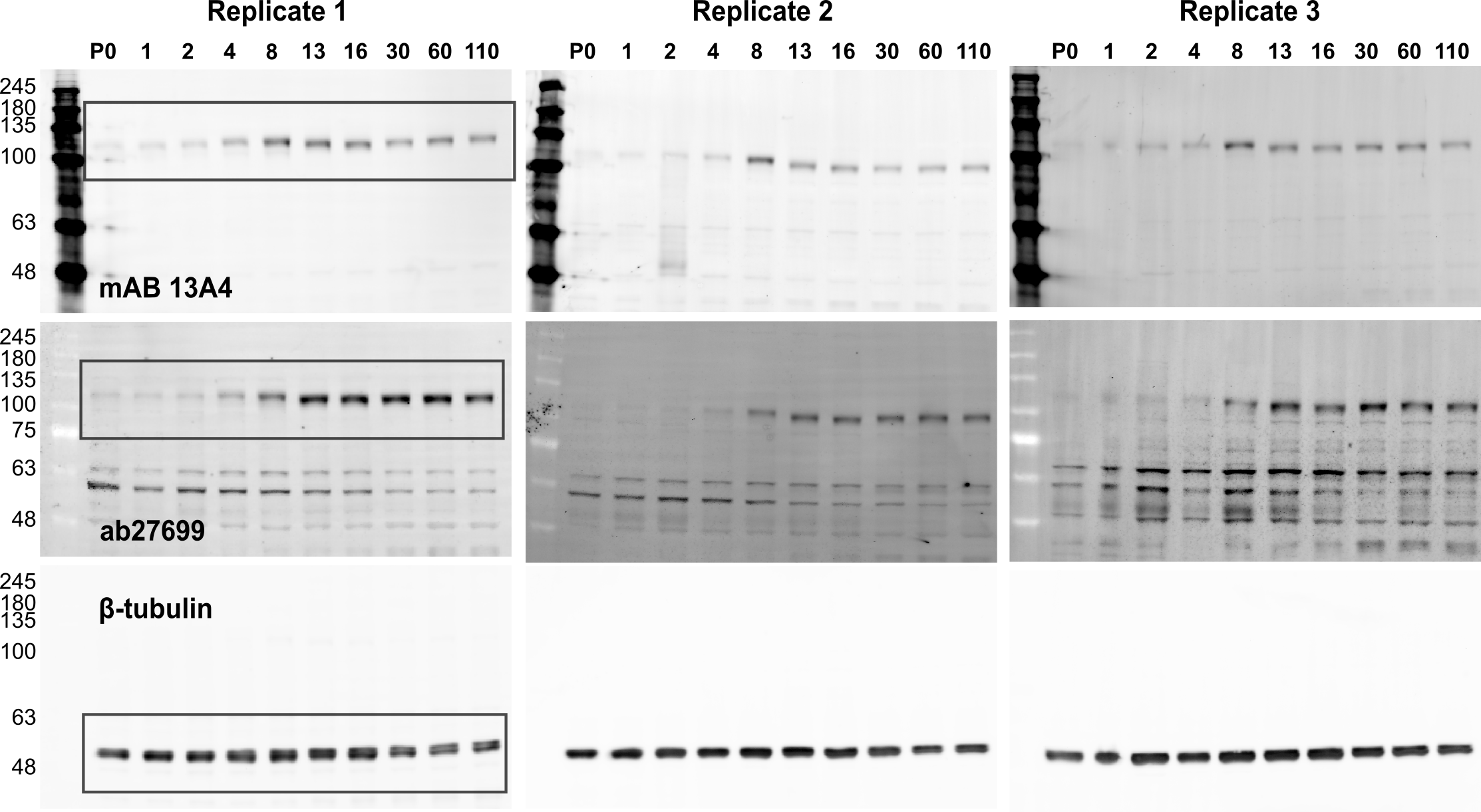

Supplement: S2 Fig — Boxes denote the parts of the images used in preparing Fig 1A. (TIF) [file pone.0274958.s003.tif]

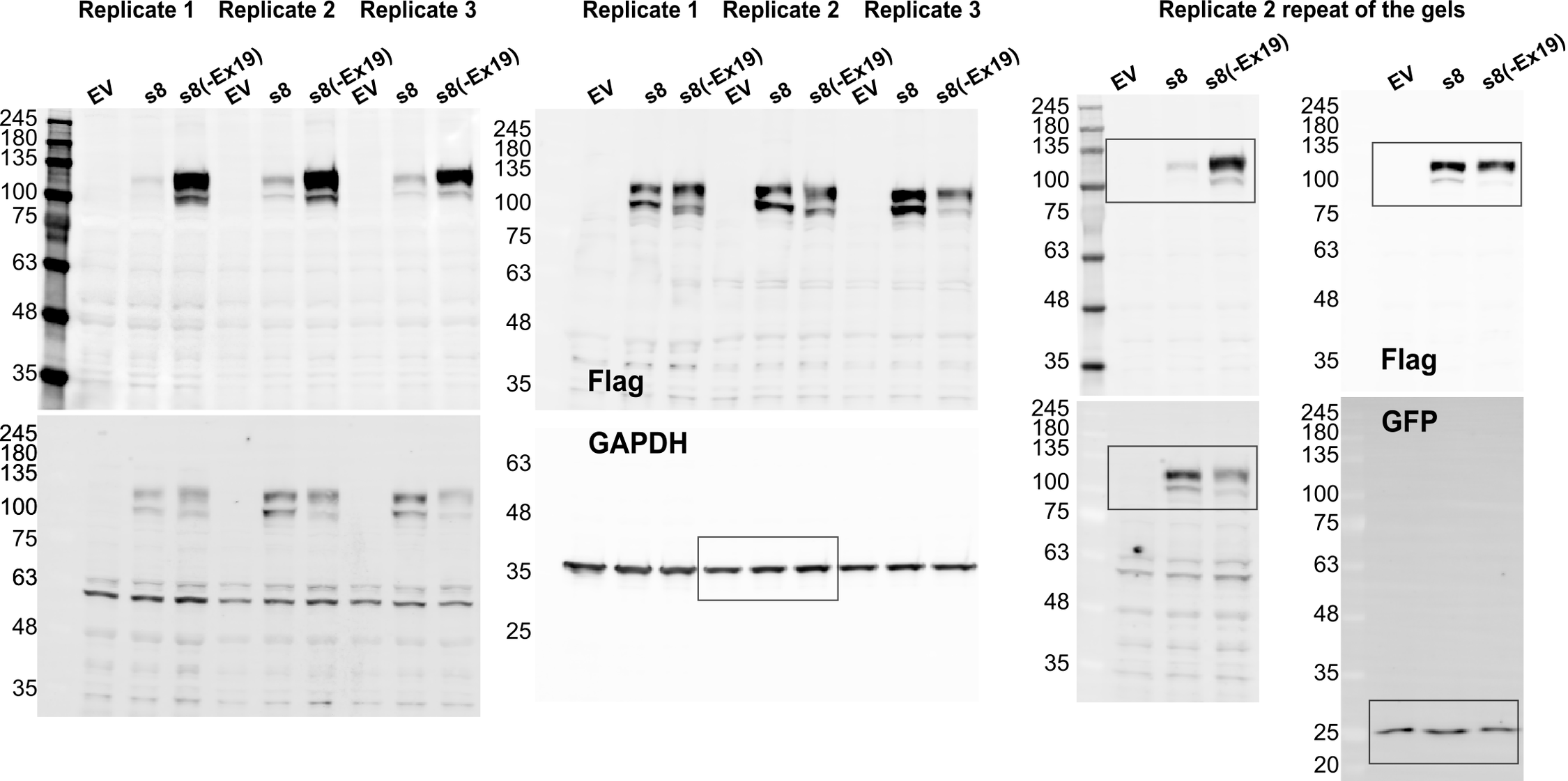

Supplement: S3 Fig — Boxes denote the parts of the images used in preparing Fig 3B. (TIF) [file pone.0274958.s004.tif]

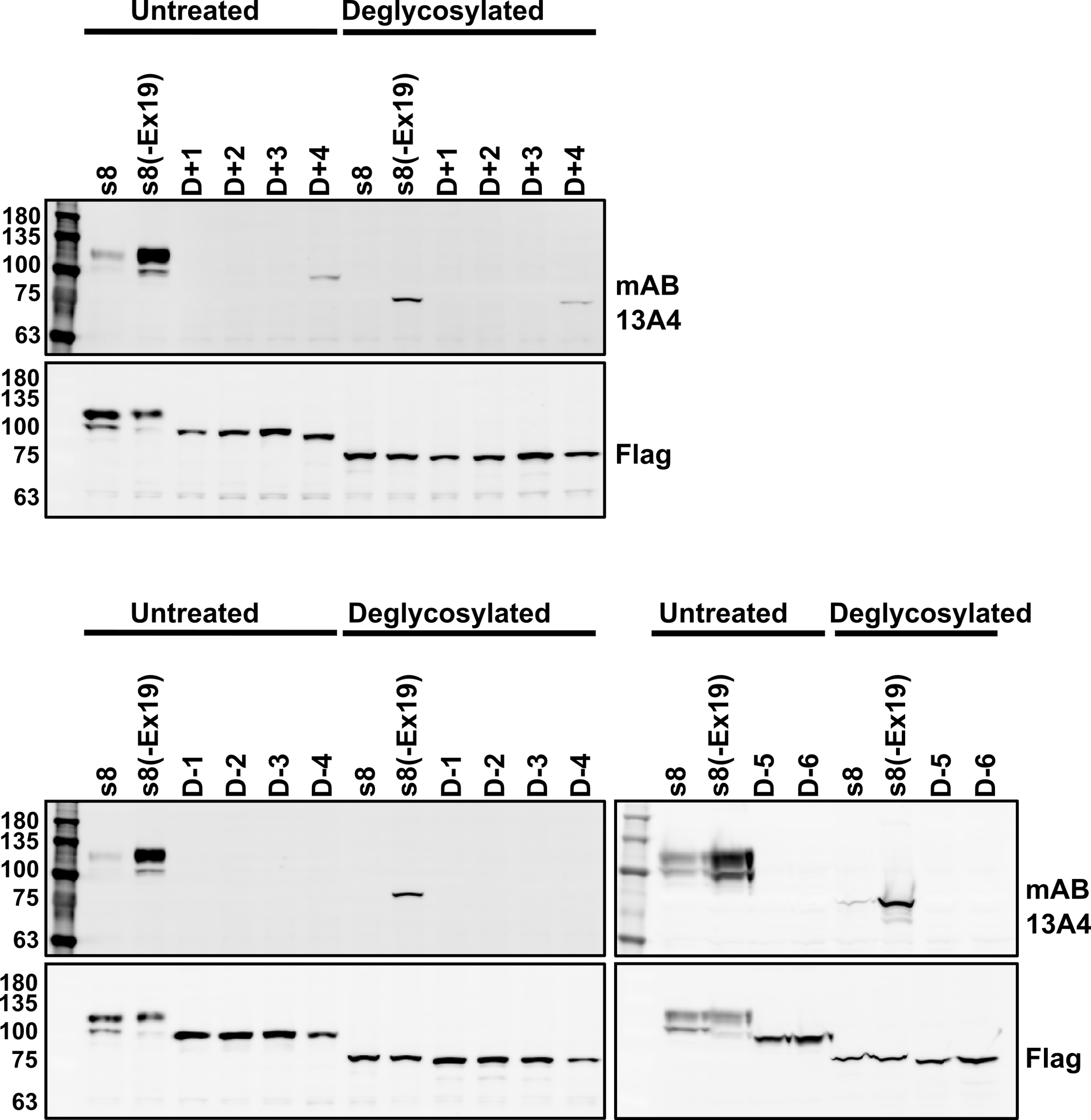

Supplement: S4 Fig — Lysates from N2a cells were treated with deglycosylation mix II (NEB) and analyzed on western blot next to untreated controls. The blots were probed with mAB 13A4 and anti-Flag antibodies as indicated. (TIF) [file pone.0274958.s005.tif]

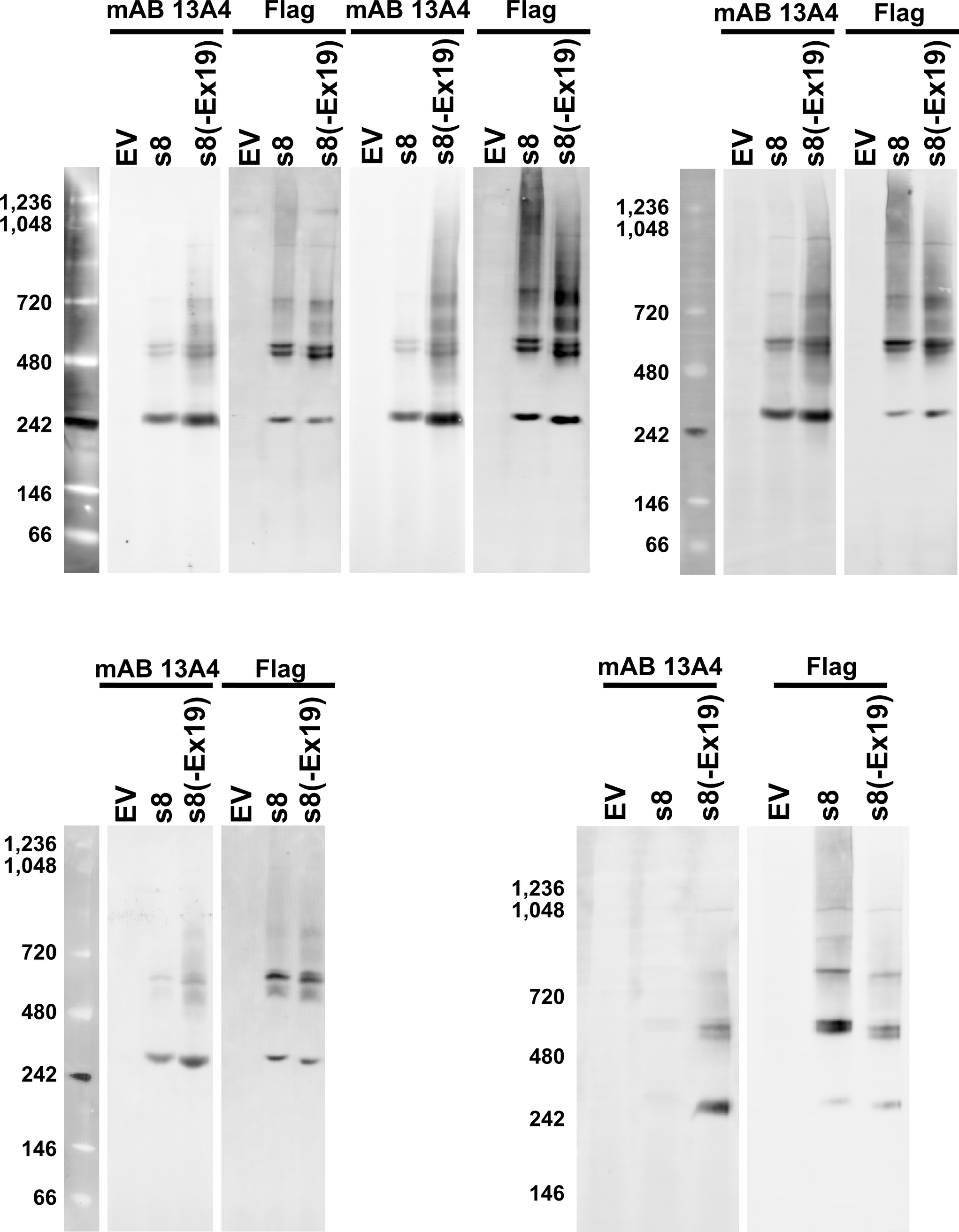

Supplement: S5 Fig — The proteins were resolved by native blue electrophoresis, transferred to PVDF membranes, and probed with mAB 13A4 and anti-Flag antibodies as indicated. Lanes containing the size standard were cut from the membranes after the transfer and stained with Ponceau S. The size standard lanes and probed membranes were imaged on Typhoon Phosphorimager (GE Healthcare). (TIF) [file pone.0274958.s006.tif]

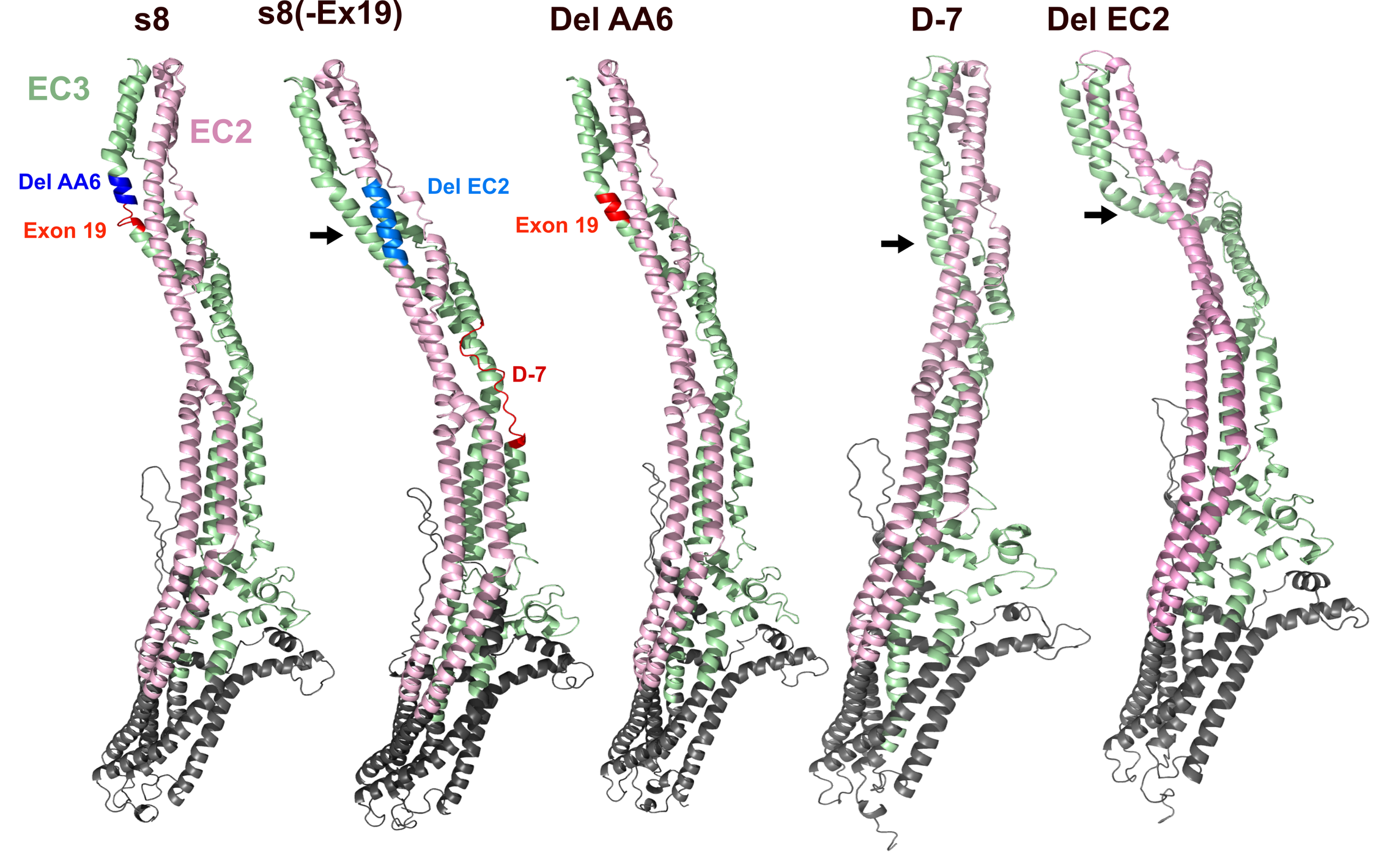

Supplement: S6 Fig — Extracellular domains 2 (EC2) and 3 (EC3) are indicated on the predicted structures by green and pink color respectively. Exon 19 and the amino acids deleted in Del AA6 are colored on the structure of the s8 isoform in red and blue, respectively. The deletions for clones Del EC2 and D-7 are colored on the structure of s8(-Ex19) in light blue and red, respectively. In structures that do not contain exon 19 arrows indicate the position of the junction between exons 18 and 20. The structures of s8 and Del EC2 have pronounced kinks near the top of the bundle when compared to the structures of s8(-Ex19). Del AA6, and Del EC2. (TIF) [file pone.0274958.s007.tif]
