# Supplementary material for: The mAB 13A4 monoclonal antibody to the mouse PROM1 protein recognizes a structural epitope
Source: PLoS One. 2022 Oct 10;17(10):e0274958. doi: 10.1371/journal.pone.0274958 (PMC9550058; doi:10.1371/journal.pone.0274958)

Original images for blots and gels

All images were captured on Typhoon Imager (GE) in fluorescence mode. Bands were detected either by the fluorescence of the secondary antibodies or by their autofluorescence (GFP and pre-stained size standards).

Fig. 2A and Supplementary Fig. 2

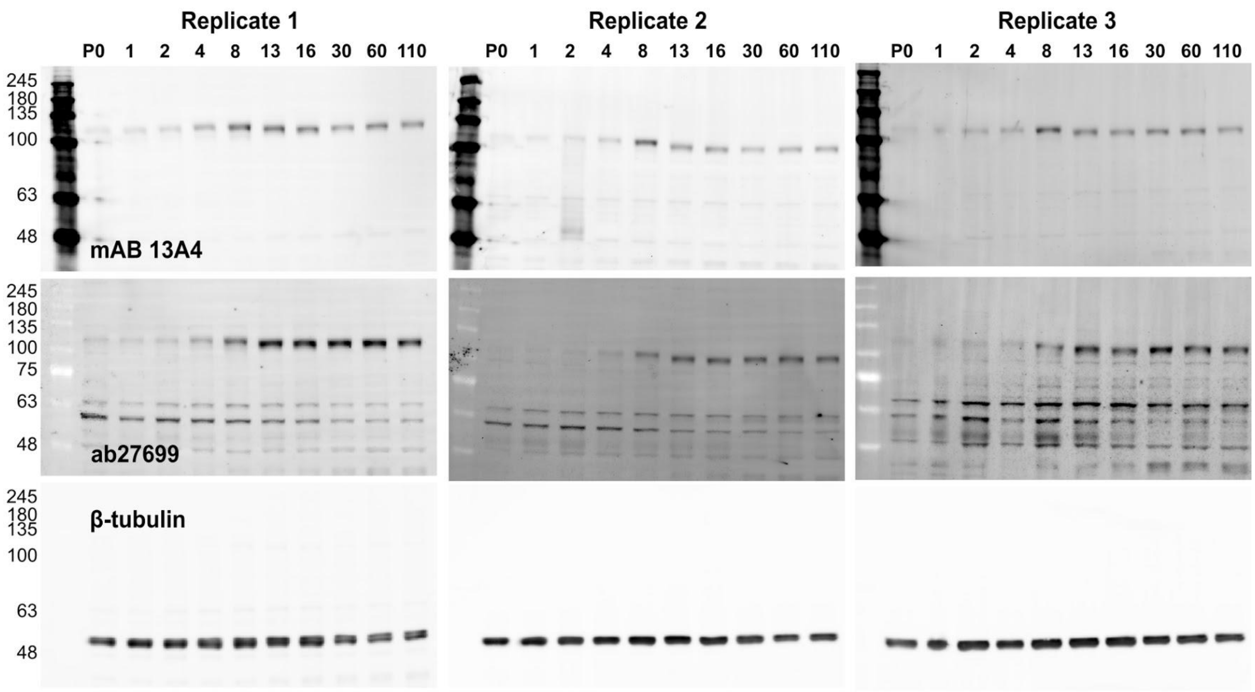

Fig. 2C

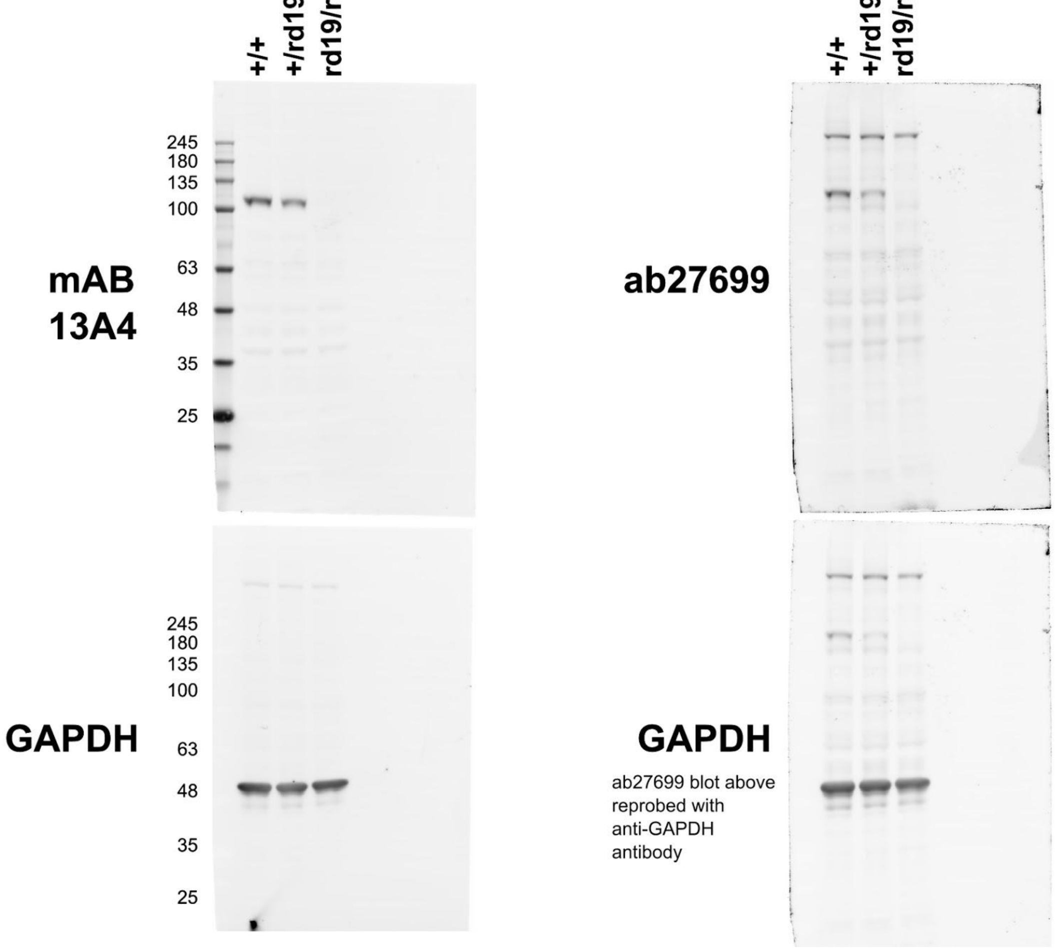

Fig. 3B and C

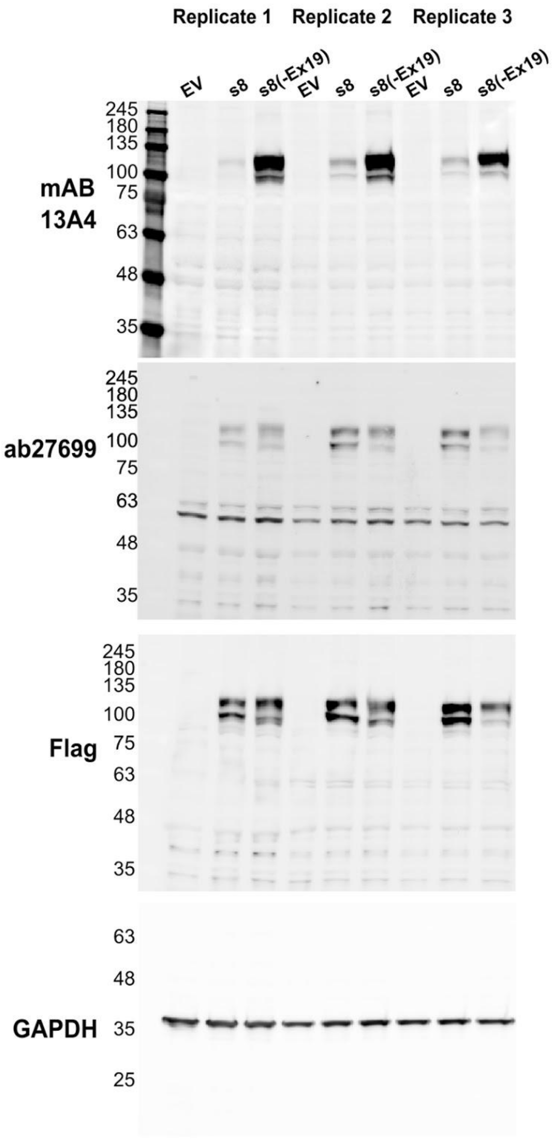

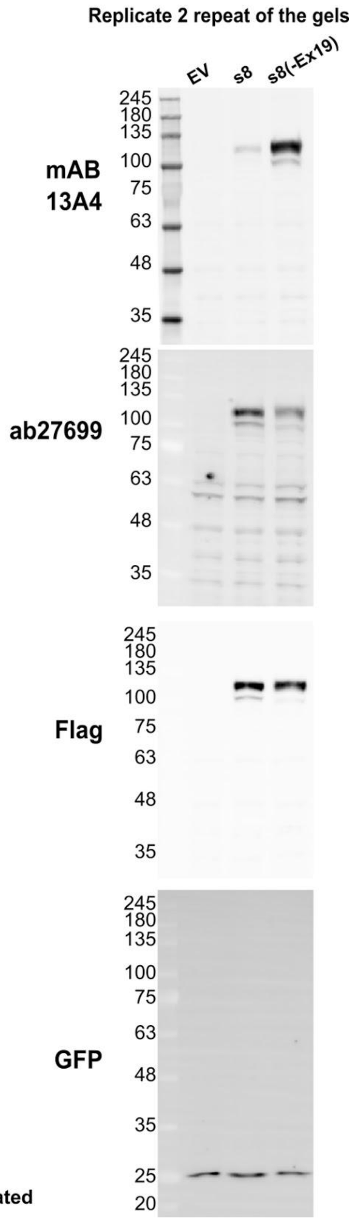

Fig. 3D

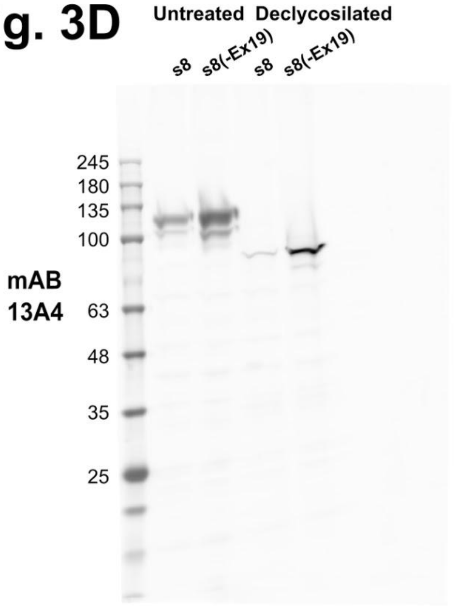

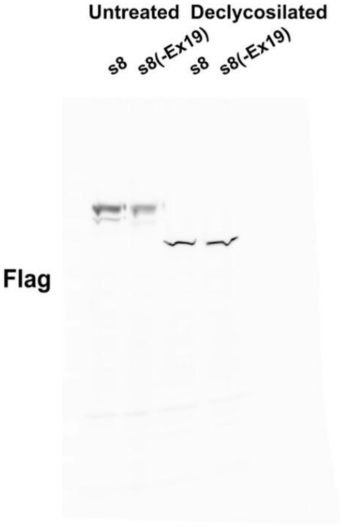

Fig. 4B

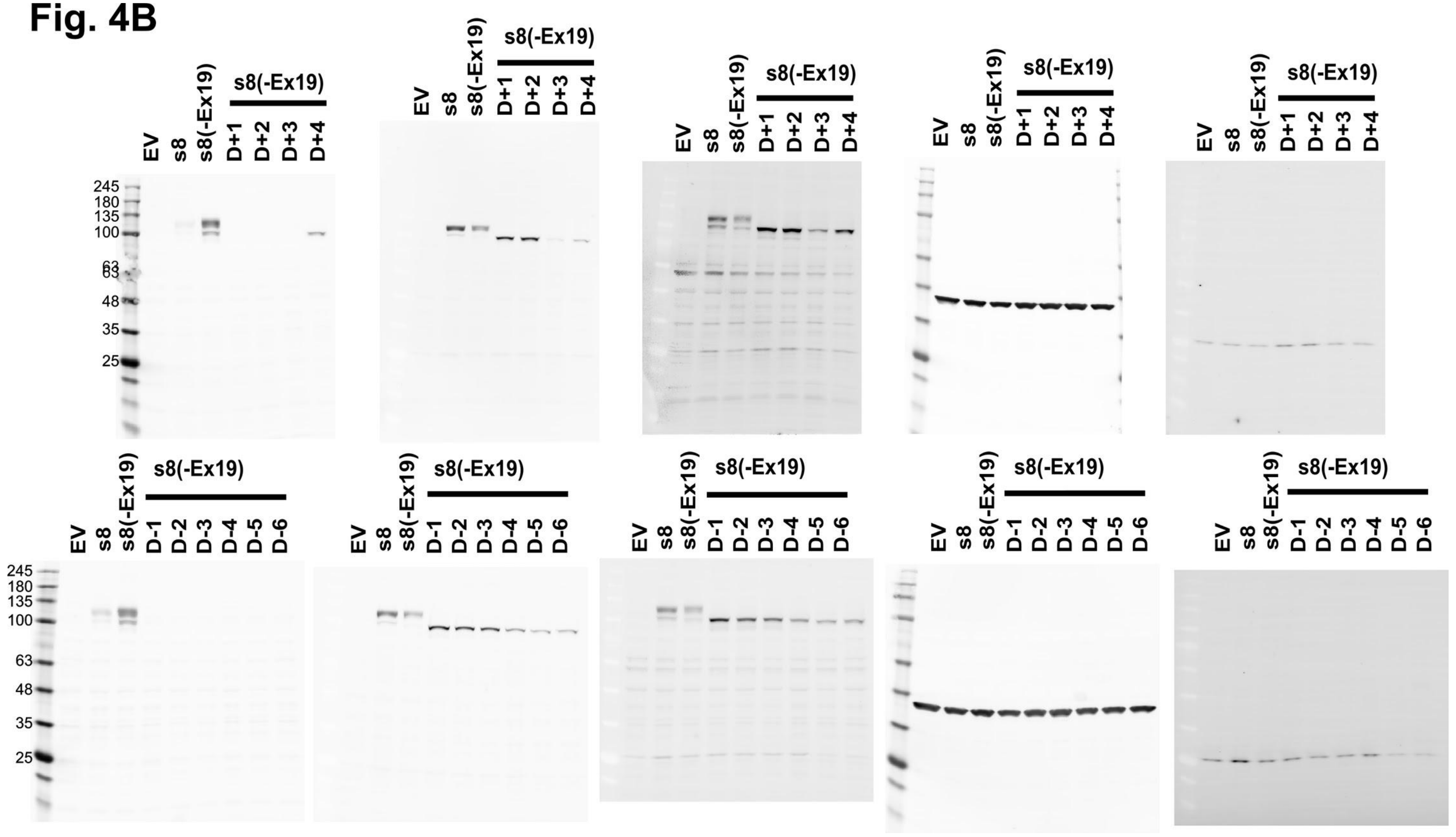
EV
s8
s8(-Ex19)
s8(-Ex19)
D+1
D+2
D+3
D+4
D-1
D-2
D-3
D-4
D-5
D-6
245
180
135
100
63
48
35
25

Fig. 5B

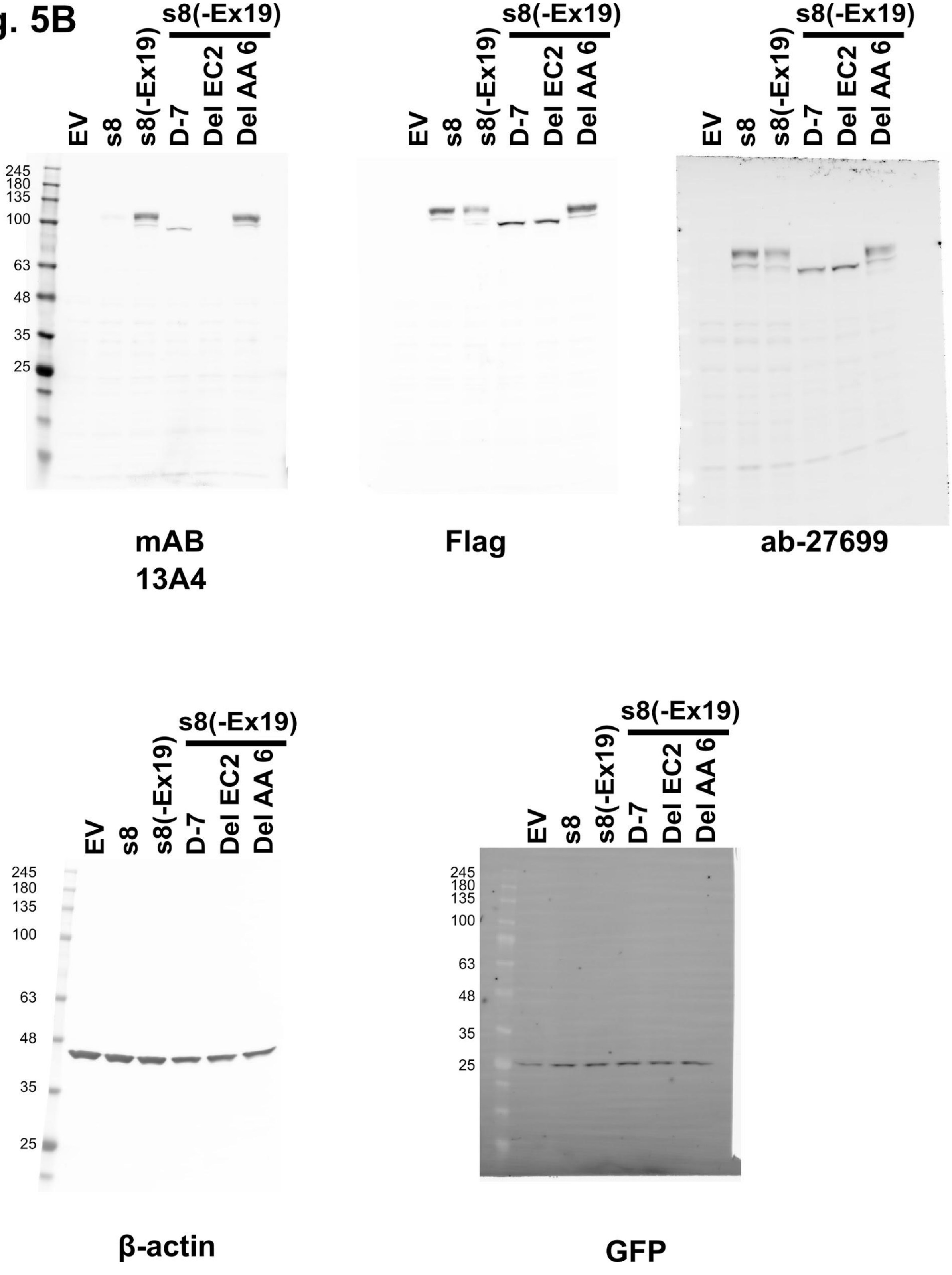
s8(-Ex19)
EV
s8
s8(-Ex19)
D-7
Del EC2
Del AA 6
245
180
135
100
63
48
35
25
mAB 13A4
Flag
ab-27699
β-actin
GFP

Supplementary Fig. 4

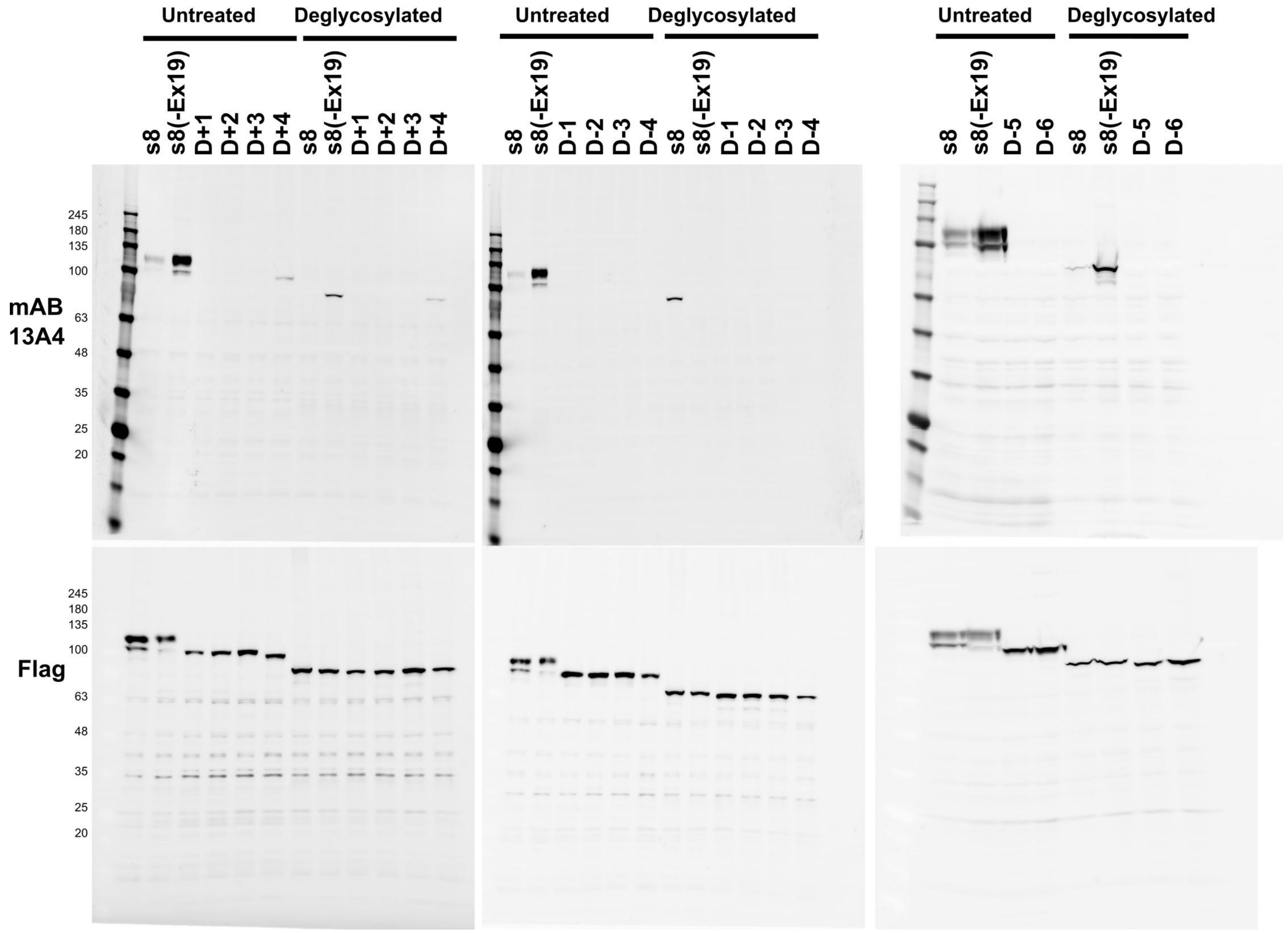

Fig. 4C and Supplementary Fig. 5

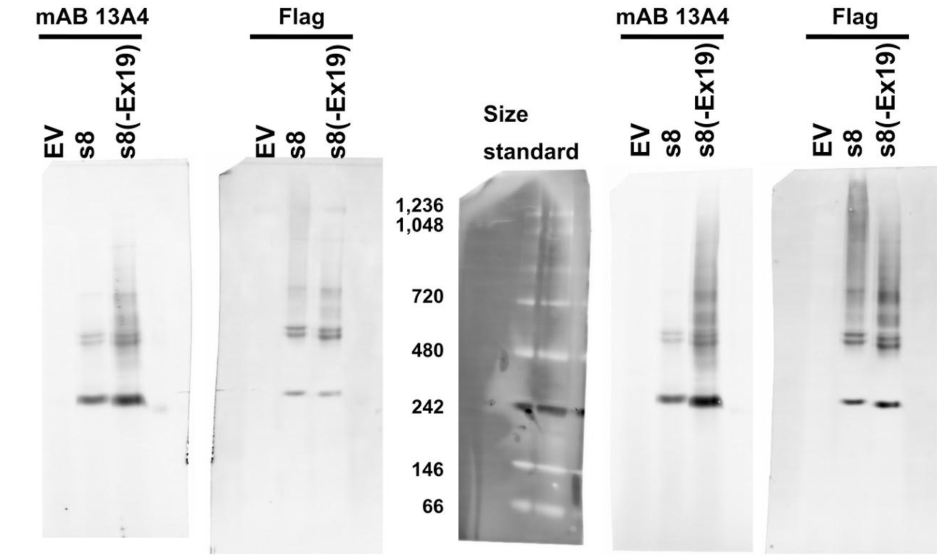

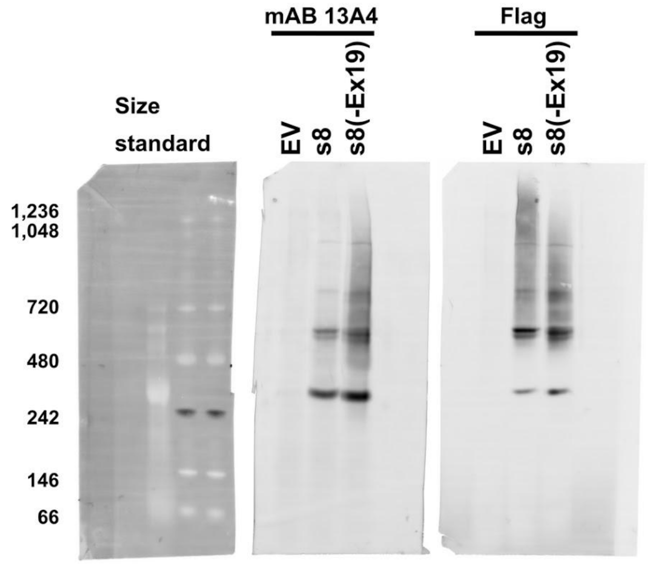

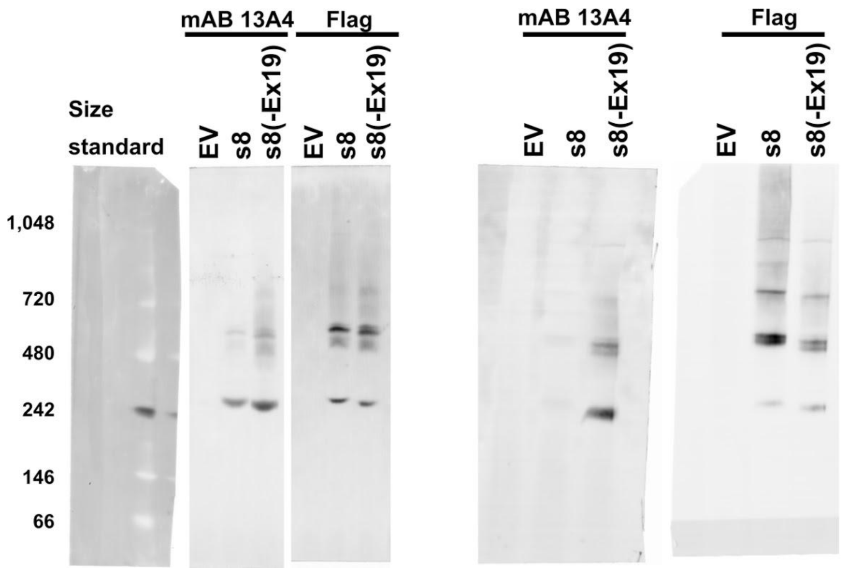

Supplement: S1 Raw images — (PDF) [file pone.0274958.s008.pdf]
